# The Impact of Geriatric Conditions in Elderly Patients with Coronary Heart Disease: A State-of-the-Art Review

**DOI:** 10.3390/jcm13071891

**Published:** 2024-03-25

**Authors:** Gonzalo Luis Alonso Salinas, Pedro Cepas-Guillén, Amaia Martínez León, César Jiménez-Méndez, Lucia Lozano-Vicario, María Martínez-Avial, Pablo Díez-Villanueva

**Affiliations:** 1 Cardiology Department, Hospital Universitario de Navarra (HUN-NOU), Calle de Irunlarrea, 3, 31008 Pamplona, Spain; amaiamtzleon@gmail.com; 2 Navarrabiomed (Miguel Servet Foundation), IdiSNA, 31008 Pamplona, Spain; lucia.lozanovicario@gmail.com; 3 Heath Sciences Department, Universidad Pública de Navarra (UPNA-NUP), 31006 Pamplona, Spain; 4 Quebec Heart and Lung Institute, Laval University, 2725 Ch Ste-Foy, Quebec, QC G1V 4G5, Canada; pedro.cepasguillen@gmail.com; 5 Cardiology Department, Hospital Universitario Puerta del Mar, Avda Ana de Viya 21, 11009 Cádiz, Spain; cesarjm91@gmail.com; 6 Geriatric Medicine Department, Hospital Universitario de Navarra (HUN-NOU), Calle de Irunlarrea, 3, 31008 Pamplona, Spain; 7 Cardiology Department, Hospital Universitario La Princesa, Calle Diego de León 62, 28006 Madrid, Spain; mariam-avial@hotmail.es (M.M.-A.); pablo_diez_villanueva@hotmail.com (P.D.-V.)

**Keywords:** acute coronary syndrome, coronary artery disease, frailty, sarcopenia, comorbidity, cognitive impairment, delirium, elderly, bleeding risk, thrombotic risk, geriatric syndromes

## Abstract

The growing geriatric population presenting with coronary artery disease poses a primary challenge for healthcare services. This is a highly heterogeneous population, often underrepresented in studies and clinical trials, with distinctive characteristics that render them particularly vulnerable to standard management/approaches. In this review, we aim to summarize the available evidence on the treatment of acute coronary syndrome in the elderly. Additionally, we contextualize frailty, comorbidity, sarcopenia, and cognitive impairment, common in these patients, within the realm of coronary artery disease, proposing strategies for each case that may assist in therapeutic approaches.

## 1. Introduction

The continuous aging of the population, coupled with advancements in prevention and treatment of cardiovascular disease, is leading to a progressively higher average age at the onset of acute coronary syndrome (ACS). This phenomenon poses a challenge, compounded by the scarcity of evidence regarding interventions in this population, a consequence of the historically low inclusion of elderly patients in studies forming the basis of current clinical recommendations. While ACS represents one of the leading causes of morbidity and mortality among older adults, geriatric syndromes are common and multifactorial clinical disorders that affect the health and quality of life in this population. Comprehensive evaluation of these syndromes in older patients with Coronary Artery Disease (CAD) allows a holistic and personalized approach, addressing not only the cardiac disease itself but also the functional, cognitive, emotional, and social conditions that may influence the management and prognosis of cardiovascular disease. Frailty, sarcopenia, cognitive impairment, delirium, and polypharmacy are the most prevalent geriatric syndromes among older adults with CAD. All of the aforementioned disorders affect the prognosis of elderly CAD patients and modify the risk/benefit ratio of different interventions, thereby increasing the complexity of their management. Hence, it is essential to understand these syndromes in order to adapt our clinical practice when they arise.

This review aims to thoroughly examine the current evidence in this field and address the influence of geriatric syndromes on CAD.

## 2. Initial Treatment of ACS in the Elderly

Older adults represent an increasing proportion of ACS patients, but they are often excluded from or under-represented in clinical trials. Moreover, older age is associated with frailty, multimorbidity, and a greater risk of both ischemic and bleeding events in ACS [1]. However, current European guidelines recommend applying the same therapeutic strategies as in younger patients, although decisions regarding how to manage older patients should be individualized based on patient characteristics [2].

The aging process is linked to heightened inflammation known as “inflammageing”. This, in conjunction with endothelial dysregulation, significantly contributes to the aforementioned elevated risk of both ischemic and bleeding events [3,4,5]. The hemostatic imbalance toward increased clotting and decreased fibrinolysis, blood stasis, endothelial dysfunction, vessel inflammation, and increased platelet reactivity may enhance thrombotic risk. In contrast, collagen and amyloid deposits in the arterial wall weaken the vessel, predisposing to bleeding. In addition, comorbidity could also further increase both bleeding and thrombotic risks, and affects pharmacokinetic and pharmacodynamic responses to antithrombotic drugs [6]. Moreover, recent studies suggest that older age is associated with a higher risk of bleeding rather than thrombotic events [1].

As bleeding causes are multifactorial and variable, an individual risk assessment should be performed. Furthermore, risk scores are only moderately accurate in predicting bleeding risk in elderly patients, with PRECISE-DAPT having better accuracy than the PARIS risk score [6]. While an age of ≥75 years represents a minor bleeding risk feature in the Academic Research Consortium (ARC) for High Bleeding Risk criteria [7] (Table 1), studies have reported major bleeding events ≥4% at 1 year in this age group.

### 2.1. Antithrombotic Therapy

In this context, some bleeding reduction strategies have been considered, tailoring drug regimens to age, body weight, renal function, prior stroke, and bleeding risk category. Common preventive measures include achieving optimal blood pressure control, avoiding non-steroidal anti-inflammatory (NSAID) drugs, and gastroprotection with proton pump inhibitors [1]. Nevertheless, current guidelines recommend a P2Y12 inhibitor in combination with aspirin after an ACS and/or coronary stenting irrespective of age [2]. Thus, prasugrel and ticagrelor are preferred in the ACS setting over clopidogrel because of their superior efficacy in randomized controlled trials (RCTs) even though they are associated with a higher bleeding risk [2]. However, elderly patients are underrepresented in these RCTs. In a substudy from the PLATO trial, a subgroup analysis of patients ≥ 75 years favored ticagrelor 90 mg twice daily over clopidogrel, as in younger patients. On the other hand, in a POPular AGE trial, clopidogrel compared to ticagrelor led to less bleeding without increasing the composite of all-cause death, myocardial infarction (MI), stroke, or bleeding. In the SWEDEHEART registry that included data of 14,005 ACS patients ≥80 years, ticagrelor provided similar efficacy to clopidogrel but increased bleeding and mortality. In the TRITON-TIMI 38 trial, compared with clopidogrel, prasugrel was associated with an increase in major bleeding in patients, with ACS undergoing percutaneous coronary intervention (PCI). Such excess in bleeding resulted in a neutral net clinical benefit in elderly patients, and because of this, prasugrel is generally not recommended in patients ≥ 75 years. The EMA and ESC guidelines recommend a prasugrel dose reduction from 10 to 5 mg daily for patients ≥ 75 years based on pharmacokinetic data. At these doses, prasugrel provides comparable efficacy and safety to clopidogrel, without clinical benefit. Nevertheless, clopidogrel-induced antiplatelet effects have a broad interpatient variability, with elderly subjects at increased risk for high platelet reactivity, a marker of thrombotic risk [1,6].

The general recommendation is to maintain up to 12 months of dual antiplatelet therapy (DAPT) with aspirin and P2Y12 inhibitor after ACS. The introduction of novel drug-eluting stents with an improved safety profile has allowed abbreviated durations. For patients with high bleeding risk (HBR), DAPT can be shortened to 1 month after elective PCI and to 3 months (or even 1 month in very HBR) after ACS, followed by aspirin or clopidogrel monotherapy [2]. On the contrary, extended DAPT beyond 12 months in older patients should be carefully evaluated or even be avoided in some cases. Moreover, for elderly patients, the latest consensus support avoiding routine P2Y12-inhibitor administration before coronary angiography in the setting of non-ST-segment elevation myocardial infarction (NSTEMI) [1].

The greatest anti-ischemic benefits of more potent P2Y12 inhibitors are seen within 30 days after ACS, and a possible bleeding reduction strategy could be the switch from a more to a less potent P2Y12 inhibitor (clopidogrel) after the acute phase. Although there are considerable number of patients who may have HPR, an age-specific analysis showed that platelet function test-guided de-escalation was not associated with a net clinical benefit in older patients [6].

**Figure 1:**
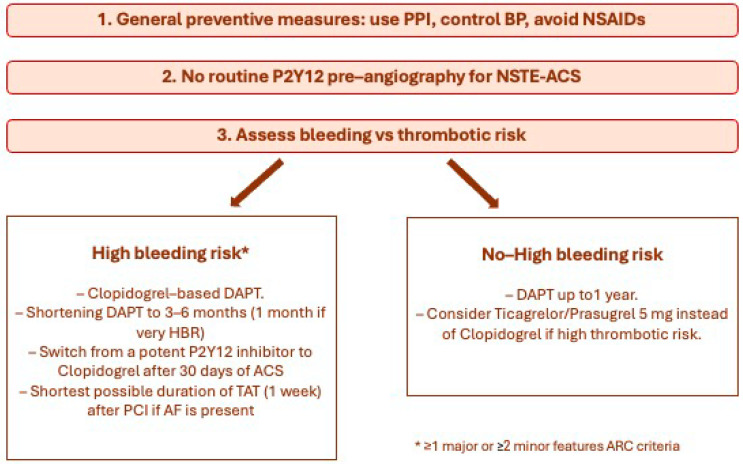
ACS antithrombotic therapy approach in the elderly. PPI: proton pump inhibitor; BP: blood pressure; NSAIDs: non-steroidal anti-inflammatory drugs; DAPT: dual antiplatelet therapy; HBR: high bleeding risk, TAT: triple antithrombotic treatment, PCI: percutaneous coronary intervention, AF: atrial fibrillation, ARC: Academic Research Consortium.

Finally, in patients with atrial fibrillation (AF) and ACS, current consensus supports dual antithrombotic therapy (DAT) with a direct oral anticoagulant (DOAC) at the recommended dose for stroke prevention and an antiplatelet agent (preferably clopidogrel) after a short period of triple antithrombotic therapy (TAT) (1–2 weeks from the acute event that can be prolonged in case of high-ischemic risk), followed by DAT up to 1 year and (D)OAC monotherapy thereafter [2]. In the elderly, the shortest possible duration of TAT is recommended after PCI if AF is present [8]. Moreover, older patients have more favorable outcomes on OAC than without, and on DOACs than on VKA. Intracranial bleeding remains lower with all DOACs compared to VKA, but a significant effect on age on increased extracranial major bleeding was observed on the higher dose of dabigatran. There was no age interaction between the rate of extracranial major bleeding and apixaban, edoxaban, or rivaroxaban, and it appeared lower with apixaban and edoxaban compared to VKA, even in older age groups [9]. Figure 1 summarizes the antithrombotic approach in elderly patients presenting with ACS.

### 2.2. Revascularization Approach

Balancing the benefits and risks of implementing invasive management in older patients presents a significant challenge. Therefore, the decision regarding the revascularization approach in older patients can be complex. Nevertheless, the evidence regarding optimal revascularization management for older patients with ACS primarily stems from recent studies conducted from 2010 onwards. This is because their inclusion was low, and in certain instances, they were explicitly excluded from the principal RCTs carried out in the late 1990s and early 2000s [10,11,12].

In the setting of ST-segment elevation myocardial infarction (STEMI), older patients were the principal population to benefit from the introduction of primary PCI (pPCI) due to the high rates of major bleeding, particularly intracranial bleeding, associated with fibrinolysis therapy [13]. This observation is substantiated by the collective analysis of the Zwolle, SENIOR PAMI, and TRIANA studies, where pPCI demonstrated superiority over fibrinolysis [14]. On the other hand, various national registries have demonstrated that, concurrently with the increased utilization of pPCI, a reduction in mortality has been observed, with older patients being the most beneficial group [15,16,17]. Even in very old patients, the performance of pPCI has been shown to be both feasible and effective [18,19]. Hence, age per se should not be an exclusion criterion for undergoing pPCI in older patients with STEMI, as recommended by the latest clinical guidelines [2].

However, the benefit of routine use of an invasive approach is not as clear in older patients with NSTEMI, with the available evidence showing contradictory results [20]. The After Eighty randomized trial demonstrated a significant benefit of an early invasive approach in older patients with NSTEMI [21]. These positive results associated with an early invasive approach persist at the 5-year follow-up, demonstrating a significant gain in event-free survival, as shown in the recently published long-term follow-up of this study [21]. Similar results were observed in the prospective observational SENIOR-NSTEMI study [22]. Conversely, in the Italian Elderly ACS, RINCAL, and MOSCA-FRAIL trials (the latter including only frail elderly patients), no differences were observed between a routine invasive treatment vs. conservative management in older patients with NSTEMI [13,23,24]. Several factors may contribute to the divergent results observed among these studies, with the burden of comorbidities and the frailty of the included patients identified as the primary factors, both associated with a worse prognosis after NSTEMI [25,26]. This information is detailed in Table 2, summarizing the key characteristics of the patients enrolled in these studies. In accordance with existing evidence, a greater burden of comorbidities and frailty entail a lower benefit derived from an invasive approach [27,28]. This underscores the need for a comprehensive assessment of older patients with ACS, including the use of geriatric scales, and tailoring management on an individual basis. Ongoing large studies will attempt to elucidate this crucial aspect [29,30]. Finally, in a selected group of patients (those with a low burden of comorbidities and no frail) presenting with ACS and having multivessel and/or left main coronary artery disease (CAD), coronary artery bypass grafting should be considered if percutaneous treatment is not feasible [31,32].

**Figure 2:**
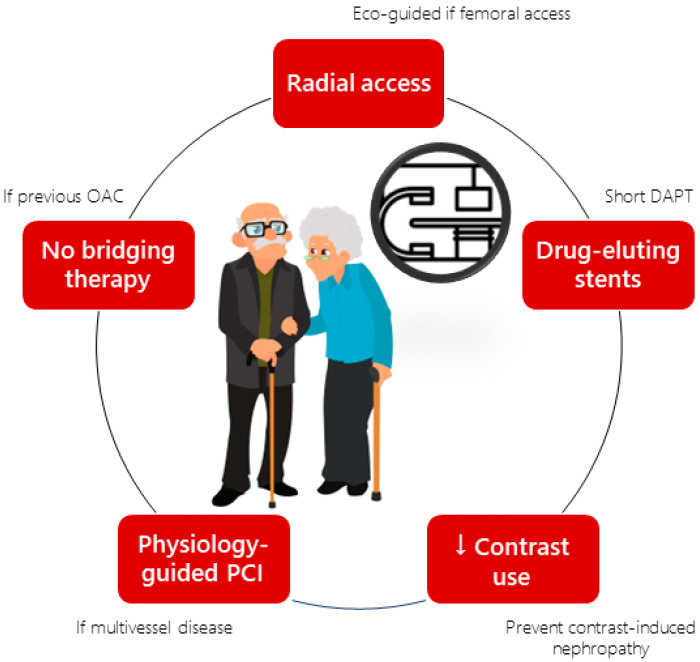
Procedural recommendations in older patients with acute coronary syndrome undergoing percutaneous coronary intervention. DAPT: dual antiplatelet therapy; OAC: oral anticoagulant; PCI: percutaneous coronary intervention. This cover has been designed using images from Flaticon.com.

In any case, in all older patients with ACS undergoing invasive management (coronary angiogram ± PCI), certain precautions should be considered due to their higher likelihood and greater impact of developing adverse events. These recommendations are summarized in Figure 2. The use of radial access should always be prioritized over femoral access given its lower complication rate [33]. In cases where radial access is not possible, ultrasound-guided femoral puncture is recommended, utilizing a micropuncture kit and small-diameter introducers [34]. For patients previously anticoagulated (e.g., those with AF), the use of bridging therapies is discouraged, as well as the use of potent intraparental antithrombotic drugs such as glycoprotein IIb/IIIa inhibitors [35]. Given the higher prevalence of chronic kidney disease (CKD), contrast use should be minimized to prevent contrast-induced nephropathy. Regarding the type of stent, drug-eluting stents are the preferred choice in all scenarios for older patients with ACS, including those where a short-term anti-thrombotic therapy (1–3 months) is desired, based on the results of various studies demonstrating their safety in this setting [36,37]. Lastly, in the presence of multivessel disease, complete revascularization guided by physiology should be performed, considering the positive outcomes observed in the recently published FIRE study compared to performing PCI solely in the culprit artery. However, whether the results of this study can be extrapolated to frail patients is uncertain, as frailty was not systematically evaluated in the FIRE trial [38].

## 3. Impact and Role of Geriatric Conditions and Comorbidity

### 3.1. Frailty

Frailty is considered as a marker of the individual’s biological age and constitutes a decrease in the physiological reserve, representing a state of vulnerability with a higher risk of adverse events [39,40]. It is prevalent in older patients with acute and chronic cardiovascular disease [41], entailing a worse prognosis in both the long and the short term [20,42].

Current guidelines recommend routinely addressing frailty and comorbidity burden in patients admitted with ACS [2]. However, frailty assessment, which are ideally conducted in the community setting, is not often performed [43]. On the other hand, several scales have been proposed to measure frailty in the ACS scenario, like the Green score, more complex, and the FRAIL and Clinical Frailty Scale (CFS), which may be of choice to assess patients’ condition in an acute setting since they are simple and time-efficient [40,44].

In ACS settings, frail patients less frequently undergo an invasive strategy, and they often receive lower prescriptions of potent antiplatelet therapies and secondary prevention drugs [45]. This may be due to a higher concern about side effects, including higher perceived morbidity and mortality [41,43,46,47].

The role of an invasive approach and revascularization in elderly frail patients with NSTEMI has been addressed. A recent RCT showed no differences in the one-year primary outcome (days alive and out of the hospital or a composite of cardiac death, reinfarction, or post-discharge revascularization) in frail elderly patients (≥4 on the CFS) admitted for NSTEMI and randomized to interventional or conservative management [48]. Likewise, in a substudy of the LONGEVOSCA registry, authors found that an invasive strategy was independently associated with better outcomes at 6-month follow-up in very elderly patients with NSTEMI, but only in those without frailty [49].

Finally, the question of whether interventions targeting frailty can improve this prognosis remains to be clarified [50]. In this context, it is important to address frailty and other geriatric conditions in a cardiac rehabilitation program as explained afterwards [43].

### 3.2. Sarcopenia

Sarcopenia is an age-related syndrome characterized by a loss of muscle mass that also involves a decrease in muscle strength and/or physical capacity [51]. The prevalence of sarcopenia is 5–15% in patients > 65 years, but it can be higher in hospitalized older adults with coronary heart disease (22.6–43%) [52,53,54]. Sarcopenia increases the risk of falls and fractures, impairs the ability to perform activities of daily living (ADLs), and contributes to lowered quality of life (QoL), higher institutionalization, and death [55]. On the other hand, the presence of sarcopenia is also associated with cardiorespiratory diseases and adversely affects the cardiovascular system, causing endothelial and vascular dysfunction. Erkan et al. found that sarcopenia was an independent risk factor for higher major adverse cardiovascular events in elderly patients with NSTEMI, and other studies also showed poor outcomes in this patient profile [56,57,58]. Moreover, a relationship between poor handgrip strength, slowing walking pace, and CAD has been observed [59,60].

Even though the current EWGSOP2 criteria are used to diagnose sarcopenia in Europe, there are other criteria, such as Asian criteria, which justifies the considerable heterogeneity in the studies and the lack of uniformity in the conducted research. This makes the extrapolation of results challenging [61,62].

However, sarcopenia is a preventable and reversible geriatric syndrome, as it can be treated. For this purpose, it is essential to consider the interrelationship with other syndromes such as cachexia, malnutrition, and frailty, requiring a comprehensive geriatric assessment.

The two main pillars for managing sarcopenia are nutrition and physical exercise. Regarding nutrition, a high-protein diet (1–1.2 g of protein/kg/day) is recommended since the requirements for older adults are higher than those for young individuals to maintain muscle mass. Furthermore, intakes should be balanced (high volumes slow gastric emptying and induce satiety), with around 15–20 g of protein recommended at each meal. It is also important to avoid fasting, and although the superiority of animal protein over plant-based protein has not been proven, the best muscle synthesis performance is achieved with protein intake after physical exercise. Other supplements such as creatine or beta-hydroxy-beta-methylbutyrate are showing promising results in this regard [63].

In the field of physical exercise, although there are no specific studies in patients with CAD and sarcopenia, the expert consensus guidelines of International Exercise Recommendations in Older Adults (ICFSR), indicates that patients should have 2 to 3 sessions per week combining resistance and power training at intensities of 40–80% of 1RM (1RM or 1 repetition maximum is the maximum weight one can lift, allowing only one repetition in that set and in a specific exercise). Functional exercises such as squats or sitting and standing up from a chair are also recommended, increasing speed and weight [64]. In conclusion, functional sarcopenia parameters such us gait speed and handgrip strength should be screened and considered prognostic and therapeutic targets in older adults with CAD to improve the detection of cardiovascular risk, better estimate the prognosis of these patients, and carry out an appropriate clinical intervention that ensures their autonomy and QoL.

### 3.3. Cognitive Impairment and Delirium

Cognitive impairment is defined as a disruption to some cognitive function such as memory. According to the DSM-5 criteria, a major neurocognitive disorder, which corresponds to dementia, requires substantial impairment to be present in one or, usually, more cognitive domains [65].

The prevalence of cognitive impairment in older adults with NSTEMI can reach up to 48% and implies significant adverse health outcomes. In older patients with NSTEMI undergoing an invasive strategy, cognitive impairment was independently associated with increased 30-day mortality and long-term all-cause mortality [66,67,68]. Moreover, there is a relationship between cognitive impairment and MI. Johansen et al. found that incident MI was not associated with a decrease in global cognition at the time of the event but was associated with faster declines in global cognition, memory, and executive function over time [69]. Therefore, post-acute MI is a risk factor for developing cognitive impairment, and preventing MI is important to preserve brain health [70].

On the other hand, delirium is a neuropsychiatric characterized by an acute change in attention and other aspects of cognition such as altered arousal, disorientation, psychosis, or mood disturbance. The incidence of delirium in Intensive Care Unit (ICU) after acute MI is around 30%, and it is associated with several poor outcomes such as a longer hospital stay, functional decline, falls, incident dementia, and higher in-hospital death [71,72,73]. The risk of delirium is determined by predisposing factors (pre-existing conditions that confer vulnerability to patients) and precipitating factors (conditions that trigger the development of this syndrome). Some studies have identified that age, cognitive impairment, alcohol abuse, sarcopenia, and depression are predisposing factors of delirium in patients with MI. Cardiac arrest, hypotension, leukocytosis, triple vessel disease, mechanical support, continuous renal replacement therapy, and respiratory failure are precipitating factors of delirium in MI [71,74].

Although there are several ways to diagnose delirium, the use of brief questionnaires based on DSM criteria is simple, useful, and quick. The Confusion Assessment Method for the Intensive Care Unit (CAM-ICU) during acute MI and the 4-AT scale in hospitalization are two of the most used scales among these patients [75,76].

Delirium prevention is also possible, being the best option to avoid its terrible consequences. The **ABCDEF** bundle component was developed in ICU for this purpose:

- **A**ssess and treat pain.
- **B**reathing trials to avoid over-sedation.
- **C**hoice of sedation avoiding benzodiazepines to perform a light sedation.
- Identify and manage **D**elirium risk factors such as a disordered sleep–wake cycle or vision/hearing impairment.
- **E**arly mobility.
- **F**amily engagement to avoid nocturnal disorientation [77].

Once delirium is established, first-line treatment involves addressing the underlying organic causes. Pharmacological treatment should only be used in cases of severe agitation, at the lowest effective dose and for the shortest duration possible. If the patient is cooperative and maintains oral intake, second-generation antipsychotics such as quetiapine or risperidone would be indicated. In cases where this is not feasible, dexmedetomidine could be considered [78], as it has been associated with a lower incidence of delirium in the ICU. Caution is advised in its use, particularly in cardiac patients, where a loading dose should be avoided due to potential side effects such as bradycardia, hypotension, and cardiogenic shock [79,80].

In conclusion, cognitive screening may play a role in risk stratification of patients with MI, and this should be considered in our clinical practice. Delirium prevention, early detection, and correct management should be implemented in ICU and hospitalization wards to avoid its consequences and preserve functional and cognitive capacities in patients with MI.

### 3.4. Comorbidity

Comorbidity is defined as the co-occurrence of several diseases. In the elderly population with ACS, comorbidity is common and significantly impacts prognosis [81]. Current guidelines recommend its routine assessment [2]. One of the most used tools to assess comorbidity is the Charlson index, a score which predicts one-year mortality in patients with multiple comorbidities [82], also in the elderly with ACS [83]. Preceding studies suggest that, as the burden of comorbidity increases, the likelihood of undergoing invasive treatment decreases. However, it is important to note that, as comorbidity increases, so do the ischemic and hemorrhagic risks [81,84]. Remarkably, in a recent retrospective study, revascularization was associated with lower 1-year mortality regardless of comorbidities in elderly patients with NSTEMI. However, this advantage diminished as comorbidity levels increased, particularly in the presence of CKD, peripheral arteriopathy, or chronic pulmonary disease [25].

CKD affects up to 75% of older adults with an ACS, conferring a worse prognosis with higher mortality and readmission rates. In fact, CKD stands out as one of the main causes of non-referral to revascularization procedures in ACS patients. Nevertheless, a comprehensive meta-analysis involving over 3000 patients revealed that revascularization, in comparison to medical therapy, entailed a lower incidence of MI in individuals with CKD [85]. Additionally, in a Spanish registry including octogenarian patients admitted for ACS, those with more severe CKD were older and showed a worse clinical profile with higher comorbidity burden and frailty. Mortality and readmission rates increased with the severity of CKD, though, interestingly, this association was only significant in patients without frailty [49].

Anemia is found in 15–20% of ACS patients, but its prevalence increases in up to 43% in the elderly subgroup of patients with ACS. Anemia is a powerful predictor of mortality in ACS after adjustment for most clinical variables and frailty [86]. Anemia plays a pivotal role in the delicate balance between ischemic and hemorrhagic risks. While elderly patients have a high ischemic risk, mainly due to their higher prevalence of cardiovascular risk factors and more complex CAD, they also present with comorbidities such as anemia or CKD, which elevates their hemorrhagic risk and reduces the likelihood of referral for invasive management [12,87]. Furthermore, as mentioned earlier, the application of bleeding risk stratification tools may result in an overestimation of risk in this population, primarily due to the underrepresentation of older patients [88,89]. Hence, individualization is crucial to determine the optimal duration of each antithrombotic therapy [20].

Polypharmacy, the concurrent use of multiple medications is also highly prevalent in this population and increases the risk of both adverse reactions and drug interactions [11]. Therefore, it is crucial to prioritize deprescribing non-essential medications to minimize the potential for drug–disease interactions that could precipitate falls, confusion, and other age-related vulnerabilities [90].

Malignancy represents the second most common cause of death globally and it becomes more prevalent with age [91]. Cancer and cardiovascular conditions are commonly associated as they share risk factors. This association can also be influenced by the state of chronic inflammation that is present in both neoplastic diseases and frailty [50,92]. Furthermore, the oncological therapies themselves may enhance the atherosclerotic process, endothelial dysfunction, thrombosis, and coronary spasm, both in active cancer patients and years after recovery [91].

Finally, it is important to remark the shared decision-making process, taking into account the preferences and goals of the patients and their families, to carefully choose best clinical management in an individualized approach in this complex scenario [93].

## 4. Secondary Prevention

Cardiovascular diseases have a significant impact on the elderly, but effective implementation of secondary prevention faces challenges due to their vulnerable characteristics and the limited scientific data. Managing traditional cardiovascular risk factors is crucial for older individuals following cardiovascular events (Table 3):

Hypertension: Older individuals should be advised to reduce salt intake and engage in regular physical exercise. A target blood pressure <140/80 mmHg should be ideally maintained, regardless of age, and may be considered below 130 mmHg if well-tolerated. However, for those aged 80 years or older or individuals with frailty, more lenient blood pressure targets are recommended [94].

Diabetes: Older patients should have a glycated hemoglobin level of 7–7.5%, with the exception of those with frail or end-stage disease [95,96].

Dyslipidemia: Target LDL-cholesterol should not differ from younger patients. Statins should be prescribed in low doses for frail patients or those at a higher risk of rhabdomyolysis. Ezetimibe has demonstrated to be a viable option to reach LDL-target goals with significant clinical benefits in older patients [97].

The treatment paradigm for dyslipidemia in patients after an ACS has undergone a transformative shift with the emergence of novel agents such as PCSK9 inhibitors, bempedoic acid, and inclisiran. Sub-group analysis from phase III outcome trials of the PCSK9 inhibitors show similar benefits in older patients with no increase in the risk of adverse events. The safety profile of bempedoic acid and inclisiran seems favorable also in this population [98]. The promising RNA interference therapy might be an advantage in those older patients with a high risk of drug interaction due to polypharmacy. Its less frequent dosing regimen offers significant advantages, enhancing adherence and potentially mitigating risks in this population [98,99].

Smoke: Smoke cessation is recommended in all patients, irrespective of age [94].

Diet: Embracing a Mediterranean-inspired diet, tailoring the diet to address specific health conditions, such as malnutrition or dysphagia [94].

Obesity: Obesity should be avoided [94].

Cardiac rehabilitation (CR) provides an optimal scenario for secondary prevention and stands as a pivotal component in cardiovascular care, correlating with reduced hospitalizations, improved QoL, and enhanced functional independence [100]. Unfortunately, older patients are frequently overlooked for referral to CR units [101]. It is essential to carefully evaluate physical frailty and functional status in order to adequately provide an individualized tailored exercise program, as frailty might be reversed [40]. Some practical tools include the Short Physical Performance Battery (SPPB), gait speed, or timed-up-and-go test, since they can assess physical capacity, detect frailty or sarcopenia, and serve as markers for monitoring their progress [102]. Not surprisingly, frail patients benefited the most in the sub analysis of the REHAB-HF trial [103]. Moreover, depression and anxiety are highly prevalent and significantly impact QoL in the older population and can also be addressed in these programs [104]. Current guidelines recommend individually adapted resistance training (RT) as part of an exercise program in patients with cardiovascular disease, although few studies have assessed the feasibility and the impact of RT in frail elderly patients with CAD [105]. RT can mitigate age-related changes in muscle function and reverse geriatric syndromes such as sarcopenia and frailty, providing safety in movement and preventing falls [106,107,108]. Although the usual way to perform CR is using weight machines [109], a home-based mobile guided CR has been demonstrated as an effective, safe alternative strategy for older adults with CAD that cannot participate in the traditional program. However, further high-quality studies are needed to investigate the impact of RT in older adults. Current evidence demonstrates the importance of a specific approach in this cohort of patients and an individually tailored training concept based on their needs.

## 5. Current and Future Perspectives

Current projections indicate an expanding aging demographic concomitant with a rise in life expectancy. Consequently, the incidence of older patients seeking treatment for ACS is expected to incrementally escalate due to the heightened cardiovascular disease risk associated with aging. Given the distinct characteristics of this demographic, characterized by an increased burden of comorbidities and the manifestation of specific geriatric syndromes, such as frailty, healthcare systems must adapt to address their unique needs, optimizing interventions and mitigating futility in specific cases. A thorough assessment of older ACS patients is imperative, encompassing the evaluation of frailty or comorbidity from the onset of hospitalization or the prevention of delirium. This strategy enables the delivery of personalized medicine, allowing interventions to be tailored to individual patient characteristics. This approach requires a multidisciplinary approach which, in any case, is not exclusive to cardiology, requiring experts in other areas such as dietetics and nutrition, physiotherapy, and geriatrics to properly individualize the management of these patients.

Moreover, there is a necessity to broaden the inclusion of ACS patients with geriatric conditions in significant clinical trials or undertake targeted studies in this demographic to address lingering uncertainties. For instance, elucidating the actual benefits of invasive management in older ACS patients with frailty or assessing the impact of short-duration DAPT following ACS. It is paramount to acknowledge that the burgeoning ACS population in this demographic will strain the already constrained resources of healthcare systems. Prioritizing the enhancement of efficiency in ACS treatment and follow-up for this population is crucial for ensuring the system’s sustainability in the future. Only through the heightened awareness of all healthcare professionals regarding the specific needs of these patients can we enhance the prognosis and QoL for older ACS patients in a sustainable manner.

## 6. Conclusions

The geriatric population presenting with ACS poses a challenge to the healthcare system due to specific characteristics that render them an especially vulnerable group. A comprehensive geriatric assessment is indispensable, since it can guide and individualize treatment based on each patient’s unique characteristics.

## Figures and Tables

**Table 1:** Academic Research Consortium for High Bleeding Risk criteria [7].

Major Features	Minor Features
Anticipated long-term oral anticoagulation	Age ≥ 75 years
Estimated GFR < 30 mL/min	Estimated GFR 30–59 mL/min
Hemoglobin < 11 g/dL	Hemoglobin 11–12.9 g/dL for men and 11–11.9 g/dL for women
Spontaneous bleed requiring hospitalization or transfusion within 6 months or recurrent bleed	Spontaneous bleed requiring hospitalization or transfusion within 12 months not meeting major feature
Platelet count < 100 × 10^9^ per liter	Chronic use of NSAIDs or steroids
Bleeding diathesis or cirrhosis with portal hypertension	Any ischemic stroke not meeting major feature
Active malignancy (excluding non-melanoma skin cancer) within 12 months	**High bleeding risk** defined by at least **1 major** or **2 minor** features.
Previous spontaneous ICH (at any time)	
Previous traumatic ICH within the past 12 months	
Presence of a bAVM	
Moderate or severe ischemic stroke within 6 months	
Non-deferrable major surgery on DAPT	
Recent major surgery or trauma within 30 days	

bAVM: brain arterio-venous malformation; DAPT: dual antiplatelet therapy; GFR: glomerular filtration rate; ICH: intracranial hemorrhage; NSAIDs: non-steroidal anti-inflammatory drug. Adapted from [7].

**Table 2:** Main randomized clinical trials evaluated the role of invasive strategy vs. conservative strategy in older patients with non-ST-elevation myocardial infarction.

Study	Population	Age, Sex	Comorbidities	Results
After Eighty ref	457 patients	84.8 years, 50.5% women	DM2: 17% COPD: 9% GFR: 53 mL/min per 1.73 m^2^ Anemia: Not available Prior Stroke: Not available PAD: 10.5% Frailty: No evaluated	Primary outcome (MI, urgent revascularization, stroke, and death): 40.6% invasive group vs. 61.4% conservative group; *p* = 0.0001
Italian Elderly ACS ref	313 patients	81.8 years, 50% women	DM2: 39.5% COPD: Not available GFR: 54 mL/min per 1.73 m^2^ Hb: 13.15 g/dL Prior Stroke: 12.5% PAD: Not available Frailty: No evaluated	Primary outcome (MI, CV rehospitalization, disabling stroke, severe bleeding, and death): 27.9% invasive group vs. 34.6% conservative group; *p* = 0.26
RINCAL * ref	251 patients	85.0 years, 50% women	DM2: 20.9% COPD: 12.5% GFR: Not available Hb: Not available Prior Stroke: Not available PAD: 3.2% Frailty: No evaluated	Primary outcome (non-fatal MI and death): 18.5% invasive group vs. 22.2% conservative group; *p* = 0.39
MOSCA-FRAIL * ref	167 patients	85.5 years, 52.5% women	DM2: 46.5% COPD: Not available Creatinine: 1.35 mg/dL Hb: 12.4 mg/dL Prior Stroke: 26.5% PAD: 11% Frailty: Clinical Frailty Scale 5/9	Primary outcome (days alive and out of the hospital): 284 days in invasive group vs. 312 days in conservative group; *p* = 0.12

* Prematurely stopped due to slow recruitment. DM2: diabetes mellitus type 2; COPD: chronic obstructive pulmonary disease; GFR: glomerular filtration rate; PAD: peripheral artery disease; Hb: hemoglobin; MI: myocardial infarction; CV: cardiovascular.

**Table 3:** Recommendations about control of traditional cardiovascular risk factors in the elderly.

Risk Factor	Recommendations
Hypertension	Target blood pressure < 140/80 mmHg, even <130 mmHg if tolerated. Lenient control if frailty or very older (>80 years)
Diabetes	Target glycated hemoglobin level of 7–7.5%. Lenient control in frail or terminal ill patients, avoiding hypoglycemia.
Dyslipidemia	Target LDL-cholesterol ≤ 55 mg/dL and >50% baseline reduction in very high cardiovascular risk patients.
Smoke	Smoke cessation
Diet	Adherence to Mediterranean diet.
Obesity	Overweight may be permitted. Avoid obesity.
